# Solution Radioactivated by Hadron Radiation Can Increase Sister Chromatid Exchanges

**DOI:** 10.1371/journal.pone.0144619

**Published:** 2015-12-14

**Authors:** Junko Maeda, Charles R. Yurkon, Yoshihiro Fujii, Hiroshi Fujisawa, Sayaka Kato, Colleen A. Brents, Mitsuru Uesaka, Akira Fujimori, Hisashi Kitamura, Takamitsu A. Kato

**Affiliations:** 1 Department of Environmental & Radiological Health Sciences, Colorado State University, Fort Collins, Colorado, United States of America; 2 Department of Radiological Sciences, Ibaraki Prefectural University of Health Sciences, Inashiki, Ibaraki, Japan; 3 Graduate School of Engineering, The University of Tokyo, Tokyo, Japan; 4 Research Center for Charged Particle Therapy, International Open Laboratory, National Institute of Radiological Sciences, Chiba, Chiba, Japan; 5 Research Development and Support Center, National Institute of Radiological Sciences, Chiba, Chiba, Japan; ENEA, ITALY

## Abstract

When energetic particles irradiate matter, it becomes activated by nuclear reactions. Radioactivation induced cellular effects are not clearly understood, but it could be a part of bystander effects. This investigation is aimed at understanding the biological effects from radioactivation in solution induced by hadron radiation. Water or phosphate buffered saline was activated by being exposed to hadron radiation including protons, carbon- and iron-ions. 1 mL of radioactivated solution was transferred to flasks with Chinese hamster ovary (CHO) cells cultured in 5 mL of complete media. The induction of sister chromatid exchanges (SCE) was used to observe any increase in DNA damage responses. The energy spectrum and the half-lives of the radioactivation were analyzed by NaI scintillation detector in order to identify generated radionuclides. In the radioactivated solution, 511 keV gamma-rays were observed, and their half-lives were approximately 2 min, 10 min, and 20 min. They respectively correspond to the beta+ decay of ^15^O, ^13^N, and ^11^C. The SCE frequencies in CHO cells increased depending on the amount of radioactivation in the solution. These were suppressed with a 2-hour delayed solution transfer or pretreatment with dimethyl sulfoxide (DMSO). Our results suggest that the SCE induction by radioactivated solution was mediated by free radicals produced by the annihilated gamma-rays. Since the SCE induction and DMSO modulation are also reported in radiation-induced bystander effects, our results imply that radioactivation of the solution may have some contribution to the bystander effects from hadron radiation. Further investigations are required to assess if radioactivation effects would attribute an additional level of cancer risk of the hadron radiation therapy itself.

## Introduction

Non-radioactive atoms can become radioactive through nuclear reactions when the atoms are hit by other high-energy particles. These radioactivations are not only observed in neutron exposure, but also in hadron radiation therapy, such as proton and carbon-ion radiotherapy [1–4]. Positron emitters and the subsequent annihilation gamma-rays generated from the atomic nuclei of the tissue during hadron radiation therapy have been investigated for the potential application of monitoring dose distribution inside the patient using positron emission tomography (PET) techniques [5–8]. Despite the fact that secondary radiation is emitted from the irradiated volume after hadron therapy, the biological effects of radioactivations have not been discussed well.

The majority of toxic effects of ionizing radiation are attributed to radiation-induced DNA damage in cells that are hit by radiation [9]. In addition to the target effects, a spectrum of radiation induced non-target effects that occur in cells not hit by radiation has been reported [10]. Radiation oncologists were familiar with radiation induced whole-body and tissue-based abscopal effects *in vivo*, which are explained with the post-irradiation induced soluble factors [11]. Such non-targeted radiation effects have been also recognized as a bystander effect, which was originally reported from *in vitro* research by Nagasawa and Little in 1992 [12]. In that paper, upon irradiation of 1% of cells in a culture with α-particles, an astounding 30% of the cells exhibited increased frequencies of sister chromatid exchanges (SCE). Several years later, experiments using media transferred from irradiated to unirradiated cells provided evidence that a soluble factor secreted into the media was contributing for the radiation-induced *in vitro* bystander effect [13]. Although the involvement of oxidative and inflammatory response is considered crucial, mechanisms of bystander effects are still under investigation [14, 15].

The main focus of this study was to investigate the potential impact of the radioactivated solution to cells. We hypothesized that if radioactivation of the solution causes DNA damage in cells that are not directly irradiated with the primary hadron radiation and if so, it implies that the effect is a part of bystander effects of hadron radiation. In order to investigate this, we exposed flasks filled with water (H_2_O) or phosphate buffered saline (PBS) to hadron radiation, and transferred the activated solution into unirradiated Chinese hamster ovary (CHO) cell cultures, then observed frequencies of SCE. Radioactivities of the solutions were measured by NaI scintillation detector to assess generated radionuclides. Solution transfer was also carried out in a delayed time point to confirm that the biological responses were dependent on the amounts of radioactivity. We used a radical scavenger to examine the role of free radicals in this effect.

## Materials and Methods

### Irradiation and radioactivity measurements

Hadron radiation experiments were carried out at the National Institute of Radiological Sciences (NIRS) in Japan. Carbon-ions and iron-ions were accelerated to 290 MeV/n and 500 MeV/n respectively, using the heavy ion medical accelerator (HIMAC) [16]. Protons were accelerated to 70 MeV using the NIRS-930 cyclotron [17]. Dose rates for carbon-ions, iron-ions and protons were set at 3 Gy/min, except for 100 Gy dose set at 40 Gy/min. These hadron radiations were exposed without a binary filter and used an entrance region, instead of Bragg Peak region containing many nuclear fragmentations [18]. Gamma-ray irradiations were carried out at a dose rate of 12.5 Gy/min using a Mark I-68A nominal 6,000 Ci ^137^Cs source (J.L. Shepherd, Carlsbad, CA).

60 mL of sterilized Milli-Q ultra pure water or PBS were filled in T25 flasks and exposed to radiation. Radioactivities (CPM) were measured by a GM counter (Aloka, Tokyo, Japan) up to 2 hours. A NaI scintillation detector (Shonan Co. Ltd. Tokyo, Japan) was used for detailed analysis where it recorded radioactivity and spectrum every 6 seconds. The best fit decay models were analyzed using a Prism6 software (GraphPad, La Jolla, CA) and R-square values were used to compare the fitness. Ion chamber (Aloka) was used for radiation dose (Gy/h).

### Cell Culture and Sister Chromatid Exchanges

Chinese hamster ovary cells (CHO10B2) [19] were graciously supplied by Dr. Joel Bedford of Colorado State University (Fort Collins, CO). Cell cultures were grown in alpha-MEM (Life Technologies, Grand Island, NY) supplemented with 10% fetal bovine serum (Sigma, St Louis, MO), antibiotics and antimycotics (Life Technologies) at 37°C in incubators at 5% CO_2_ and 100% humidity. Cellular synchronization was carried out as previously described [20]. 5 mL of cell culture medium containing mitotic cells was transferred into new T25 flasks and kept in the incubator. After 2-hours of incubation time, more than 95% cells were in G1-phase of cell cycle at the time of the experiments. Flow cytometry analysis confirmed the cell cycle positions. 1 mL of irradiated or unirradiated solution was added immediately to the flask or at 2 hours post exposure. Cells were incubated with 10 μM bromodeoxyuridine for two cell cycles, and the second post treatment metaphase cells were harvested with colcemid treatment for 6 hours before fixation. To verify radical scavenger effects, dimethyl sulfoxide (DMSO) (final 1% v/v) was added to cell culture 30 minutes before radioactivated solution was transferred. Differential staining was carried out using standard fluorescence plus Giemsa staining methods [12, 21, 22]. The number of SCE was scored in 50 cells for each data using a Zeiss Axiophot (Carl Zeiss, Jena, Germany) microscope equipped with a Exi Aqua cooled CCD camera (Q-imaging, BC, Canada). A single person scored all SCE without coding. Because the chromosome number of CHO cells has variation (21 on average), the data were presented as the mean number of SCE per chromosome.

### Statistical analysis

Statistical analyses were performed by Prism6 software. Correlation between radioactivities of water and PBS and between radioactivities and SCE inductions was determined using the Pearson’s correlation test. All experiments were carried out at least three times, and each data was represented in terms of mean and standard errors of the mean (SEM). The statistical comparison of radioactivities and SCE frequencies between experimental conditions was performed using a two-way ANOVA test. Statistical comparison of mean values of SCE between different exposed doses (or radioactivities) was performed using a one-way ANOVA test followed by Tukey’s multiple comparisons analysis for each dose. Differences with a p-values of <0.05 were considered to indicate a statistically significant difference. Dose-response curves were analyzed using non-linear regression, and comparisons of the curves among different hadron radiation types were done using an extra sum-of-squares F test for their slopes and intercepts.

## Results

### Radioactivity in solution

**Fig 1:**
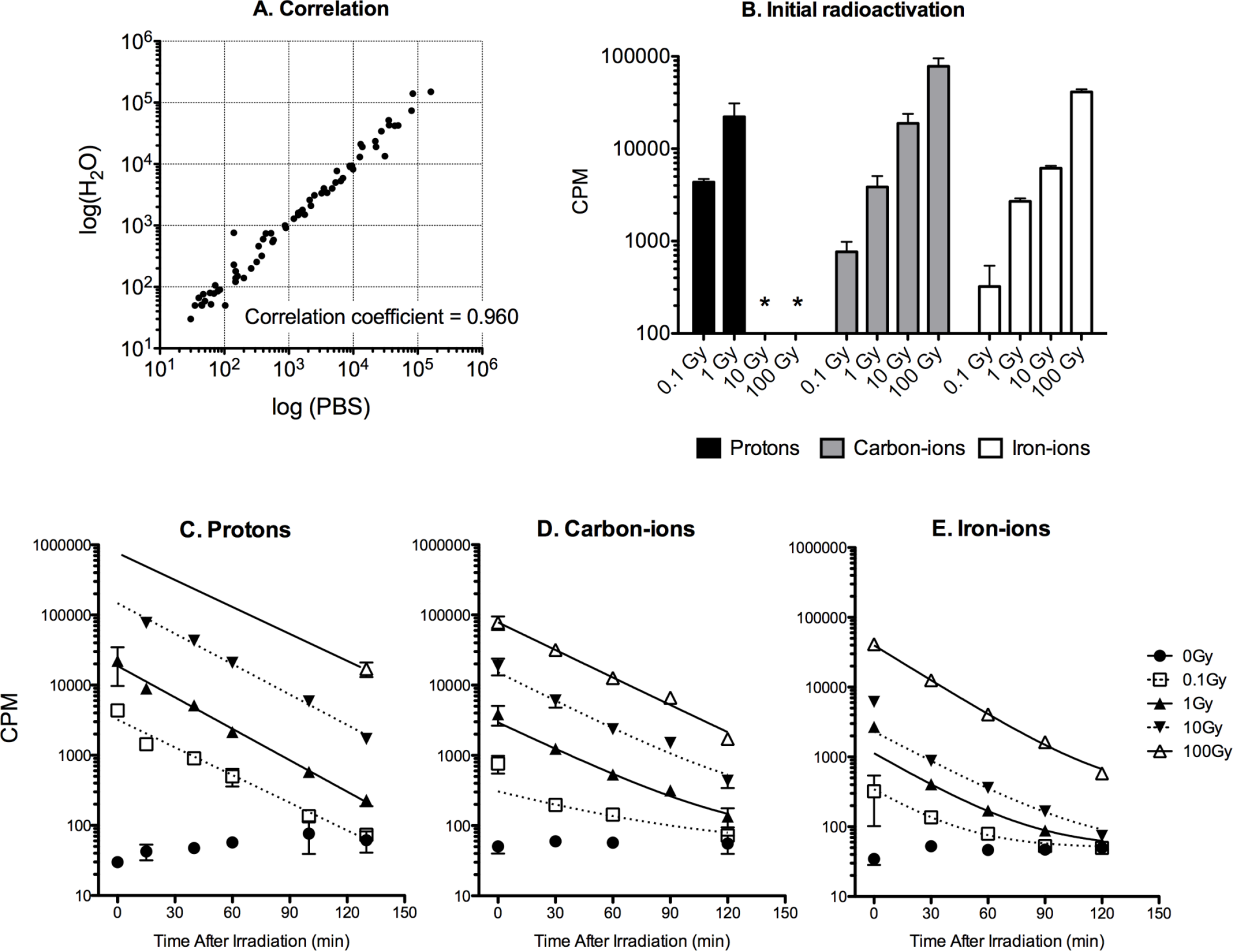
Radioactivation of the solution after different types of hadron radiation exposure. Radioactivities were measured as counts per minute (CPM) by GM counter. **(**A) Comparison of activation of H_2_O and PBS. (B) Initial radioactivation per exposed doses. The * indicates that more than 10 Gy of proton exposure induced higher than GM detector’s upper limit. (C-E) Time course of radioactivities after (C) proton, (D) carbon-ion, and (E) iron-ion exposures. Lines were fitted with one-phase exponential decay curves with half-lives of approximately 20 minutes. Experiments were carried out at least three times, and error bars indicate SEM.

0.1 Gy to 100 Gy of hadron radiation were able to activate 60 mL of sterilized water or PBS in flasks in a dose dependent manner. Radioactivities were measured as counts per minutes (CPM) by GM counter. Radioactivities between water and PBS were not significantly different (p = 0.391) and showed a nearly one to one correlation (R^2^ = 0.960, Pearson test) (Fig 1A). The amount of activation per exposed doses was the strongest in protons, followed by carbon-ions and iron-ions (Fig 1B). Immediately after exposure, more than 10 Gy of proton exposure induced radioactivities higher than the GM detector’s upper limit. Therefore, the radioactivities of 10 Gy and 100 Gy immediately after exposure were based on the other dose trends. The 100 Gy of proton exposure was estimated to induce approximately 1,000,000 CPM by GM counter, which was approximately 1.5 mGy/h by ion chamber measurement. The radioactivities based on every 15–40 minute time measurements using GM counter were fit with one-phase exponential decay models with similar slopes for all different doses and types of hadron radiation exposures, with the exception of 0.1 Gy of carbon-ions (Fig 1C and 1D). Half-lives calculated from the decay for these particle exposures were approximately 20 minutes, for example, 10 Gy of protons, carbon-ions, iron-ions showed half-lives of 20.7, 21.9 and 20.8 hours, respectively.

**Fig 2:**
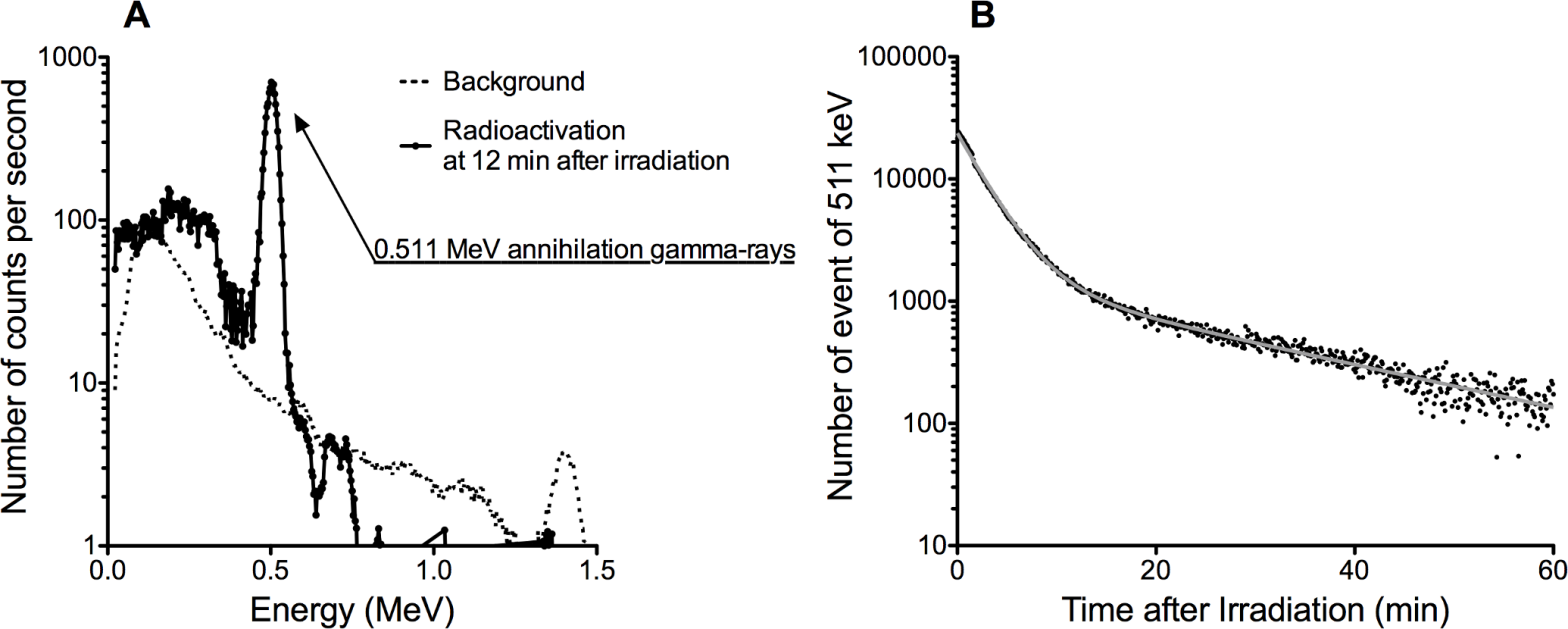
Radioactivation after 100 Gy of proton exposure to PBS measured by NaI scintillation detector. (A) Energy spectrum of NaI detector. A clear peak of annihilation gamma-rays at 0.511 MeV is shown. (B) Time course change of measured count rates of 511 keV gamma-rays. Data was fitted with three-phase exponential decay model with half-lives of 2 min (93%), 10 min (3.5%), and 20 min (4.5%).

To identify potential radionuclides after radioactivation, we used NaI scintillation detector with the ability to record gamma-ray spectra for energy spectra and counts. Fig 2A shows the result of energy spectra and counts of gamma-rays after 100 Gy proton exposure to PBS, 12 minutes after irradiation. We observed a clear peak of 511 keV, which corresponded to the annihilation of positrons. No other significant peaks of higher energy gamma-rays within the 1.5 MeV range of the detector were observed. Fig 2B shows the time variation of measured count rates of 511 keV gamma-rays from the NaI scintillation detector measurement. From actual gamma-ray counts up to 1 hour post irradiation, we compared several models including one-phase, two-phase and three-phase exponential decay models. One-phase exponential decay gave a half-life of 3 minutes (R square = 0.895). Two-phase exponential decay gave half-lives of 2 min (93%) and 15.5 min (7%) (R square = 0.948). We found that the three-phase exponential decay model fit best (R square = 0.996) where the half-lives were approximately 2 min (93%), 10 min (3.5%), and 20 min (4.5%). We assumed the existence of at least three radionuclides generated by the proton irradiation, likely corresponding with three positron emitters; ^15^O (half-life 122 sec), ^13^N (half-life 9.97 min), and ^11^C (half-life 20.4 min). However, due to our experimental limitations from time differences between irradiation and measurement, we could not validate the existence of positron emitters with shorter half-lives.

### SCE induction by radioactive solution

**Fig 3:**
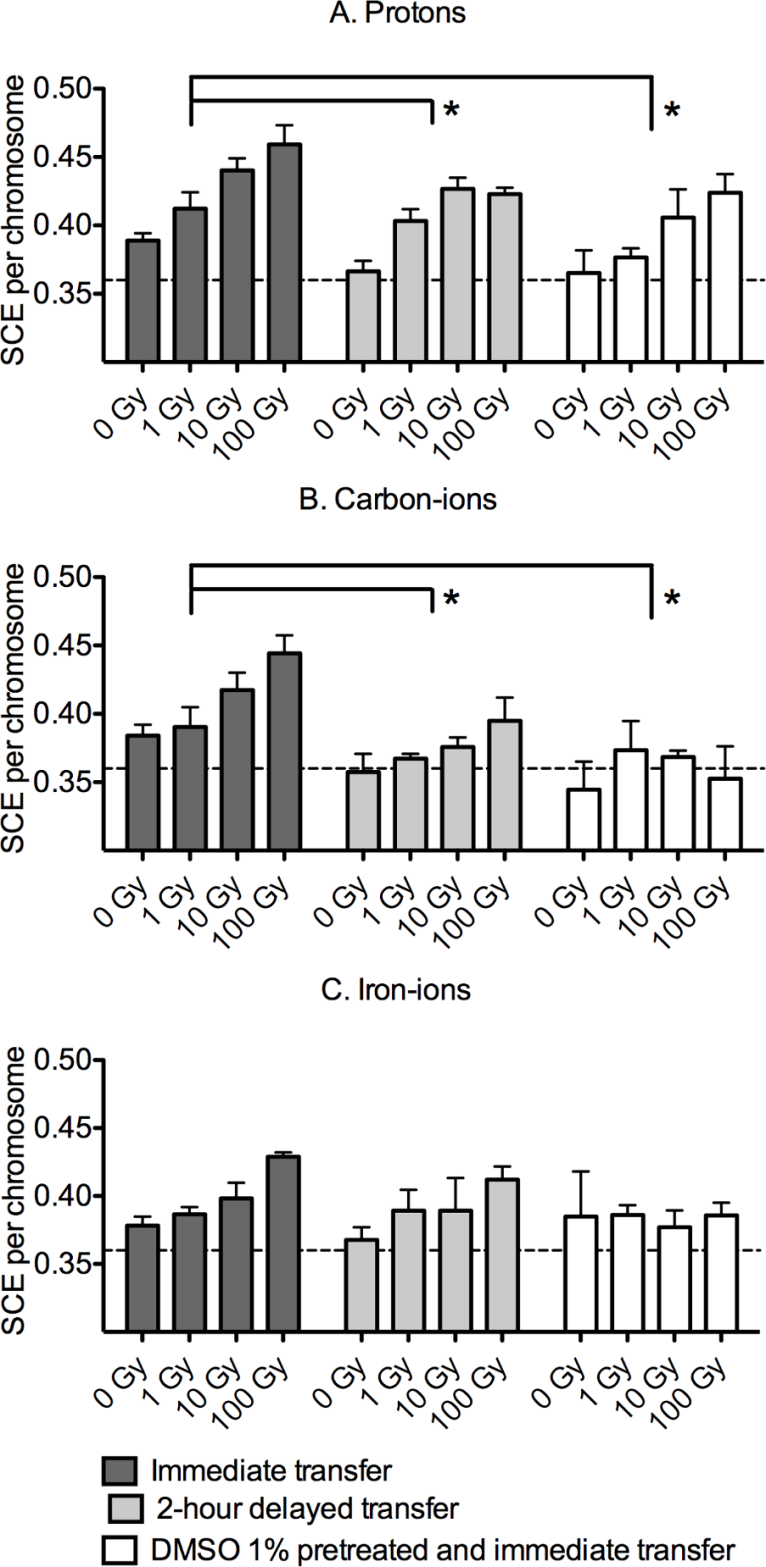
SCE induction from solution exposed to hadron radiation. (A) Protons, (B) carbon-ions and (C) iron-ions exposures are shown. Dashed line indicates non-solution transferred control values. The data and error bars are presented as the mean ± SEM of SCE per chromosome collected from water and PBS experiments. The * indicates significant differences between treatment groups by two-way ANOVA.

Exposure of CHO cells to radioactivated solution were accomplished by adding 1 mL of radioactivated water or PBS immediately or 2 hours after irradiation to the 5 mL cell culture medium. Compared to the level of SCE induction of non-solution transferred control (0.36 per chromosome), the addition of 1 mL of unirradiated water or PBS did not significantly change SCE frequency (0.37 per chromosome) (Fig 3). We did not observe statistically significant differences between radioactivated water and PBS for SCE induction (p = 0.142). Therefore, SCE induction by radioactivated water and PBS in the different experimental conditions were shown together in Fig 3. In the results of exposure to solution immediately after radioactivation, the SCE induction increased with the exposed dose of each hadron radiation type (Fig 3). Significant differences were observed in the SCE inductions of 10 Gy and 100 Gy of protons, and 100 Gy of carbon and iron ions compared to their 0 Gy levels (p<0.05). In proton and carbon-ions groups, significantly lower SCE frequencies across four radiation doses were observed in a 2-hour delayed transfer condition compared to immediate transfer condition (p = 0.007 and p<0.001, protons and carbon-ions, respectively) (Fig 3). DMSO pre-treatment suppressed the mean SCE frequencies across four radiation doses in the immediate transfer group at significant levels (p = 0.004 and p<0.001, proton and carbon respectively). Similar trends, but with no significant level, were observed in the 2-hour delayed and DMSO treatment groups for iron-ion exposure (Fig 3), where the weakest activation per exposed dosed among the different radiation types was observed (Fig 1).

**Fig 4:**
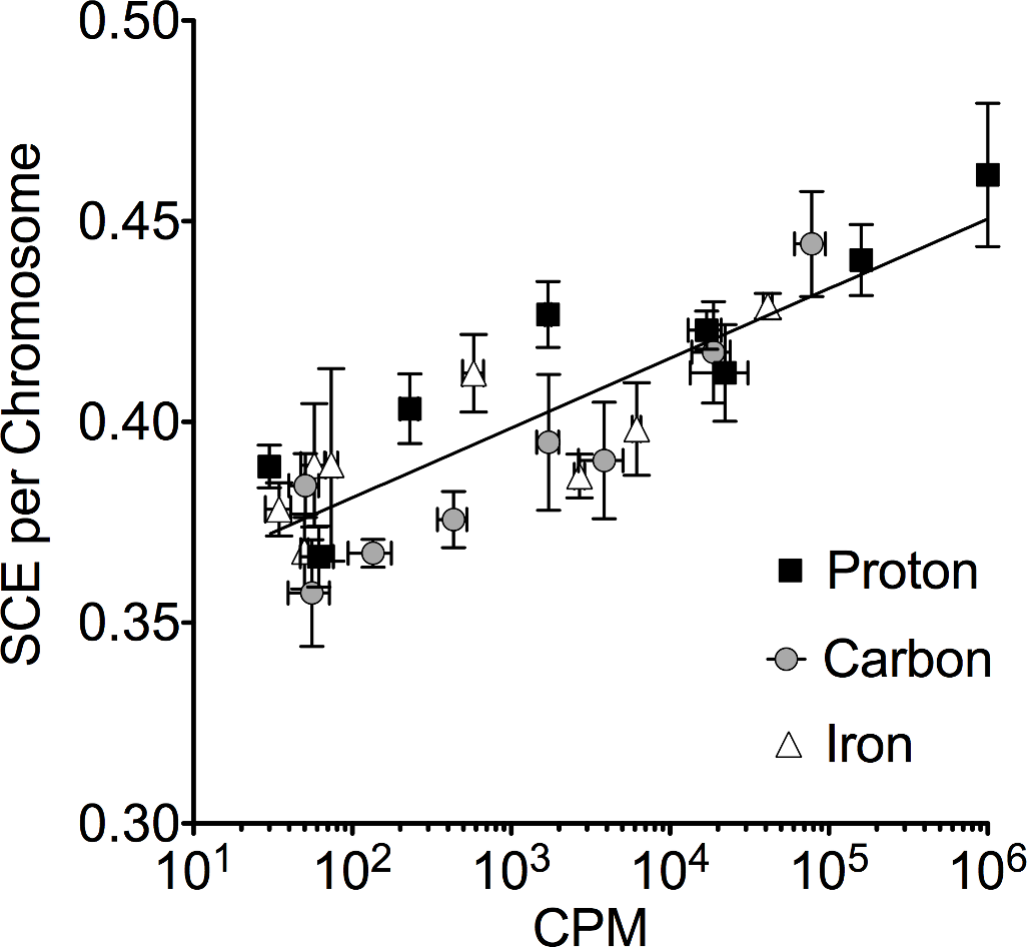
SCE induction from radioactive solutions. Radioavtivities (CPM) are at the time of solution transferred. Pooled SCE data induced by water and PBS activated by different hadron radiation types were plotted. Data were fit with semi-log line (R square = 0.722). Observed SCE = 0.017 x log(CPM) + background SCE.

SCE induction of immediate or 2-hour delayed solution transfer data (Fig 3) was plotted against radioactivities (CPM) at the time that the solution was transferred (Fig 4). When the best fit semi-log lines between the radioactivities and SCE inductions for each hadron radiation type were compared, no significant differences were observed among parameters of each line (p = 0.161). Therefore, pooled SCE data induced by water and PBS activated by all three hadron radiation types were plotted in Fig 4. A positive correlation between the amount of radioactivities and SCE inductions regardless of hadron radiation type was observed (R^2^ = 0.391, Pearson test). SCE induction was highest with 0.46 per chromosome when the solution was exposed to an approximately 1,000,000 CPM of protons (100 Gy). This was significantly increased compared to the control of 0.37 (p<0.01).

### No SCE induction by photon radiation exposed solution

**Fig 5:**
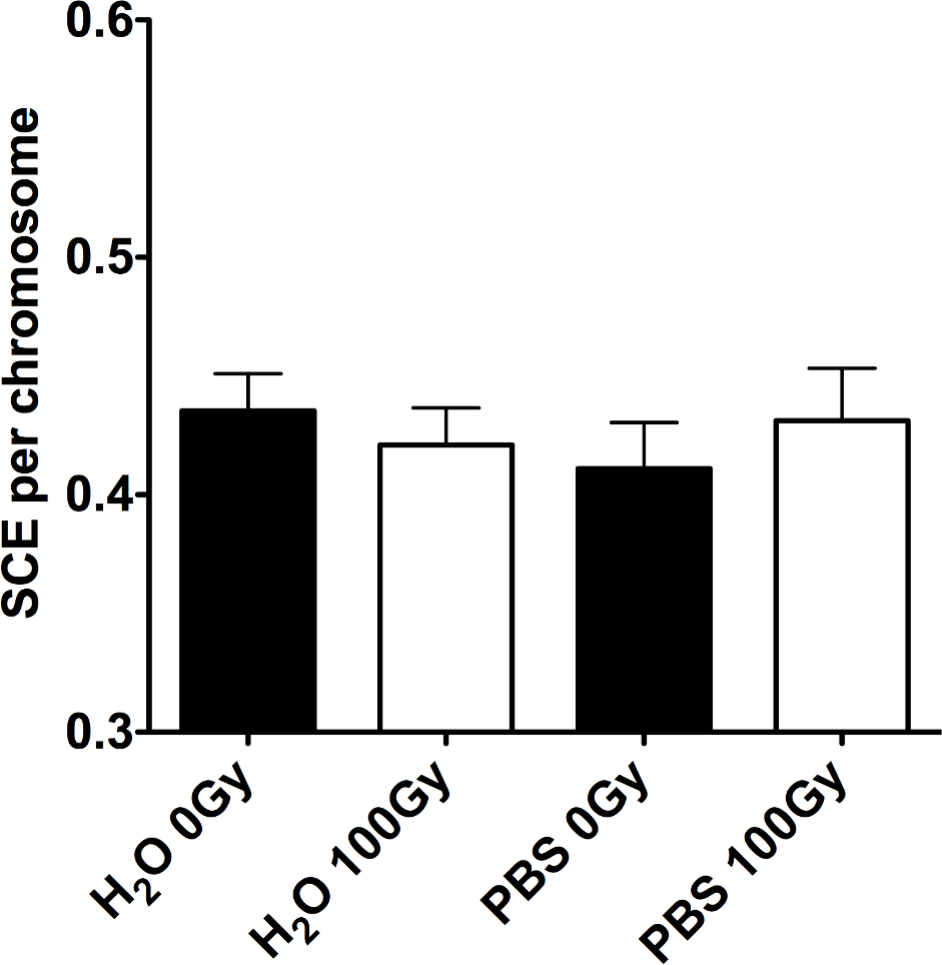
SCE induction from gamma-ray exposed solution. No significant difference was observed in the mean SCE frequencies among the samples by one-way ANOVA (p = 0.799). Experiments were carried out at least three times and error bars indicate SEM.

For the gamma-ray exposure, due to insufficient energy of 661 keV gamma-rays of ^137^Cs to generate radioactivation, no SCE induction from exposed solution was expected. Water and PBS were exposed to 100 Gy of gamma-rays to analyze SCE induction using the same protocols as those used for the hadron particle exposures. We confirmed no significant SCE induction in the samples of water or PBS exposed between 0 Gy and 100 Gy of gamma-rays (p = 0.799) (Fig 5). Slight increase of background SCE values compared to hadron radiation experiment might be associated with different lots of FBS because the experiments were conducted in two different institutes.

## Discussion

The objective of this study was to determine whether the radioactivation of water or PBS causes DNA damage in cells that are not directly irradiated with the primary radiation. In the present study, we detected the potential DNA damage from radioactivation using the frequencies of SCE.

From a physics perspective, radioactivation by proton and heavy ion beams in water as a tissue-equivalent target have been investigated [23–25]. We observed the proton exposure induced radioactivation in PBS having half-lives of 2, 10, and 20 minutes with 0.511 MeV gamma-rays (Fig 2). Those values strongly suggested the generation of ^15^O, ^13^N, and ^11^C. Other researchers have also reported that the presence of these three radionuclides in water irradiated with proton beams [24]. Based on the result of no different amounts of generated radioactivites between water and PBS after hadron radiation exposure (Fig 1), the main targets of nuclear reactions could be estimated as hydrogen or oxygen atoms. As reported in the previous studies of protons [24, 26], nuclear reactions of ^16^O nuclei, such as ^16^O(p, X)^11^C, ^16^O(p, pn)^15^O, ^16^O(p, alpha)^13^N reactions may generate these positron emitters (^11^C, ^15^O and ^13^N). Beta+ decay of those radionuclides emits a positron that annihilates with an electron, generating two gamma-rays with 0.511MeV.

The amount of radioactivation per exposed doses among proton, carbon- and iron-ions depended on an order of fluence (Fig 1). For ions heavier than protons, like the carbon and iron beams, radioactivation in solution is expected to be more complex than proton reactions. In addition to the nuclear fragmentation of water atoms, the heavy ion’s nuclear reactions also produce various lighter ions during their path through the projectile fragmentation, which has complicated the analysis of radionuclide product measurements [18, 23, 25]. In our study, the half-lives of activations based on GM counter were similar among proton, carbon- and iron-ions with approximately 20 minutes (Fig 1). This indicated that heavy ions may likely generate ^11^C, however, it is highly possible that many other radionuclides are also produced not only by the target fragmentation but also by the projectile fragmentation in the heavy ion’s nuclear reactions.

Radioactivation effects on polymers, including polystyrene (C_8_H_8_)_n_ of the cell flasks used in our study, have been reported [24, 27]. Although we cannot rule out the effect of radioactivation in cell culture flasks, we estimated that it accounted for a very small effect. This is because the reaction cross section of polystyrene is much lower than water molecules, and the radioacivation of polymer targets were smaller than water targets in the previous studies [24, 27].

Sister chromatid exchange assay is a test for the detection of reciprocal exchanges of DNA between two sister chromatids of a duplicating chromosome [28]. We observed that radioactivated solution induced SCE in a radioactivation dose dependent manner in CHO cells (Fig 4). From the estimated radionuclides, we assume that observed induced SCE by radioactivated solution could be explained by annihilated gamma-rays from positron emitters. The SCE exchange process presumably involves DNA breakage and repair, and elevated SCE frequencies are widely used for a biomarker for genotoxic stress [29, 30]. One limitation of our study is the endpoint to measure DNA damage induced by radioactivated solution as there is little known about its molecular basis and the consequences of the SCE [28]. However, compared to other cytogenetic assays such as chromosome aberration assays, the sensitivity of damage detection by SCE is high [31]. Furthermore, SCE induction has been utilized to detect radiation-induced target and non-target effects including bystander effects [12, 28, 31]. Therefore, we considered that radioactivation in solution could induce biological effects, including DNA damage.

In our study, pre-treatment of DMSO, a well-established radical scavenger, suppressed the SCE induction in CHO cells cultured in solutions that were irradiated with protons and carbon ions (Fig 3), suggesting that the SCE induction was a free radical mediated process. It is well known that ionizing radiation induced DNA damage is caused by either direct or indirect effect [32]. In each process, ejected electrons directly break chemical bonds that result in DNA strand breaks and distortions, or produce free radicals from surrounding water molecules that indirectly react with the DNA molecule [32]. It is likely that the DMSO effect seen in the present study was a modulation of the indirect effect from annihilated gamma-rays.

The DNA damage responses observed in our study may happen in cells unirradiated directly with primary hadron beams and subsequently irradiated directly with the positron annihilation gamma-rays when the cells are located near the radioactivated solution. This can be an effect observed in bystander cells. The bystander effect in radiation biology is known as a different biological effect that manifests in bystander unirradiated cells remaining within an irradiated cell population or occurs between different population even though medium transfer [10]. Importantly, the SCE induction and DMSO modulation have been also observed in the radiation induced bystander effects [12, 33]. This is involved in cell-to-cell communications or signaling from the irradiated and unirradiated cells [12, 14], and there is no doubt that reactive oxygen species [15], gap junctions [34, 35] and paracrine soluble signals are associated with this pathway [13]. Furthermore, there are many reports about bystander effects with low energy photon radiation [36, 37], while radioactivation is not caused by low energy photon radiation as seen in our results (Fig 5). Due to these differences, we are not proposing that radioactivation can explain major pathways of bystander effects. However, the effects of radioactivated solution might contribute to a part of bystander effects of hadron radiation exposure, and it could be significant in irradiation experiments with high energy radiation.

Whether there are potential health effects of such radioactivation in humans after hadron radiation therapy remains unclear. Importantly, all cells in the target volume would be traversed by many protons/several carbon ions during radiotherapy. It will be of great interest to determine if radioactivation-induced DNA damage would play an additional role in cancer risk after hadron radiation therapy. In conclusion, radioactivation of the solution exposed to hadron radiation induces DNA damage which may contribute to part of bystander effects. Considering the case where the secondary ionizing radiation emitted from irradiated volume after hadron radiation causes biological effects, the effects of radioactivation on the genome in irradiated patient should be further discussed.
